# Supplementary material for: Enhancement of an organic–metallic hybrid charring agent on flame retardancy of ethylene-vinyl acetate copolymer
Source: R Soc Open Sci. 2019 Mar 20;6(3):181413. doi: 10.1098/rsos.181413 (PMC6458352; doi:10.1098/rsos.181413)
Supplement: The structure characterization of hybrid [file rsos181413supp1.docx]

**Supporting Information**

**Enhancement of** **an organic-metallic hybrid charring agent on flame retardacy of EVA**

**Bo Xu****^*^,** **Wen Ma, Lushan Shao, Lijun Qian^*^, Yong Qiu**

*School of Materials Science and Mechanical Engineering,* *Beijing Technology and Business University,* *Fucheng Road 11, Beijing 100048, PR China*

*Beijing Key Laboratory of Quality Evaluation Technology for Hygiene and Safety of Plastics, Beijing Technology and Business University, Fucheng Road 11, Beijing 100048, PR China*

FTIR spectra of OTCA and OTCA@ZnO are presented in Figure S1. For OTCA, the peak at 3012 cm^-1^ was associated with the stretching vibration of CH_3_ from γ-aminopropyltriethoxy silane group. Peaks at 2925 cm^-1^ and 2797 cm^-1^ were attributed to the stretching vibration of CH_2_ from γ-aminopropyltriethoxy silane and ethylenediamine groups. Peaks at 1586 cm^-1^ and 1512 cm^-1^ were the characteristic absorption of triazine ring. The absorption bands at 1028-1005 cm^-1^ and 813 cm^-1^ were corresponded to Si-O and Si-C, respectively. It was worth noting that the absorption band for C-Cl at 850 cm^-1^ was not presented in the spectrum of OTCA, which indicated the Cl atoms attached on the triazine ring had been totally replaced. For OTCA@ZnO, the absorption bands for CH_2_ (2929 cm^-1^), triazine ring (1586 cm^-1^ and 1512 cm^-1^), Si-O (1083 cm^-1^) and Si-C (809 cm^-1^) could also be observed. Compared with OTCA, some obvious difference appeared in the FTIR curve of OTCA@ZnO. The strong absorption peak at 3427 cm^-1^ arose corresponding to Si-OH and the peak at 3012 cm^-1^ for CH_3_ disappeared, which indicated that the hydrolytic reaction of γ-aminopropyltriethoxy silane group occurred completely. The peak at 1083 cm^-1^ was smoother due to the hydrolysis reaction of triethoxy silane. Besides, a new peak at 434 cm^-1^ appeared corresponding to Si-O-ZnO, which revealed the condensation reaction of hydroxyl groups took place.

The chemical structure of OTCA@ZnO was further confirmed by ^1^H NMR, ^13^C SSNMR and ^29^Si SSNMR. As shown in Figure S3, the chemical shifts at 168 ppm and 76 ppm were assigned to the *C* atoms of the triazine ring. The chemical shifts at 44 ppm, 26 ppm and 14 ppm were attributed to the *C* atoms of the ethylenediamine, N-*C*H_2_-C and C-*C*H_2_-Si groups from γ-aminopropyltriethoxy silane, respectively. For the ^29^Si SSNMR spectrum, two signals at -54 ppm and -57 ppm could be observed, which were assigned to the *Si* atoms of *Si*-O-ZnO and *Si*-O-*Si* respectively. All of these characterization results proved the successful preparation of OTCA@ZnO.





**Figure S1.** FTIR spectra of OTCA and OTCA@ZnO.





**Figure S2.** ^1^H NMR spectra of OTCA@ZnO.





**Figure S3.** ^13^C NMR spectra of OTCA@ZnO.





**Figure S4.** ^29^Si NMR spectra of OTCA@ZnO.
